# Alessandro Moretta and Transporter Associated With Antigen Processing (TAP) Deficiency: On Giant's Shoulders

**DOI:** 10.3389/fimmu.2019.02404

**Published:** 2019-10-15

**Authors:** Jacques Zimmer

**Affiliations:** Laboratory of Innate Cellular Immunity and Chronic Inflammation, Department of Infection and Immunity, Luxembourg Institute of Health, Esch-sur-Alzette, Luxembourg

**Keywords:** TAP deficiency, natural killer cells, HLA class I, cytotoxicity, antibodies

## Abstract

The laboratory hosting me for my Ph.D. described in 1994 the first human cases of TAP deficiency in two siblings with recurrent bacterial airway infections and a negative Human Leukocyte Antigen class I (HLA) serotyping. At this time, it became clear that natural killer (NK) cells interact with HLA class I molecules which inhibit them. Inhibitory receptors were postulated, and Alessandro Moretta was the first to generate monoclonal anti-human NK cell antibodies that bound to such molecules, which he characterized in detail (Killer Immunoglobulin-like receptors—KIR). Natural killer cells from healthy donors preferentially kill targets with absent HLA class I molecules (“missing self” concept), whereas we observed that the NK cells from the TAP-deficient patients were hypo-responsive and did not lyse the HLA class I-negative leukemia cell line K562. Moreover, they were not very active in antibody-dependent cellular cytotoxicity assays. To address the question if such NK cells would express KIR or not, my thesis supervisor requested the anti-KIR antibodies from Alessandro Moretta, who was kind enough to provide us generously with aliquots. It turned out that the NK cells from the TAP-deficient individuals expressed most of these inhibitory receptors normally. We then had the privilege to receive almost every new antibody generated in the Moretta lab and to complete the phenotypic studies of the NK cells from our patients. I had the great chance to meet Alessandro Moretta at several occasions. He deeply impressed me each time and strongly influenced my way of thinking.

## Introduction

It is likely that most scientists meet, especially during the early phase of their career, more advanced colleagues that guide them and leave a deep impression on them for the rest of their life. My case was no exception, as I was lucky enough to find two of such mentors, namely my Ph.D. thesis supervisor, Dr. Henri de la Salle, and Professor Alessandro Moretta.

In 1995, after my medical studies in Strasbourg, France, and 2 years working at an Emergency Department, I realized that I would not want to continue spending my lifetime under such conditions, and decided to give a new orientation to my career by acquiring a Master Degree in Immunology, a field in which I always had been interested. I found an internship position at the Blood Transfusion Center in Strasbourg, in the lab of Daniel Hanau and Henri de la Salle. They had described in 1994 two siblings with a very low cell surface expression of HLA class I molecules (negative serotyping), chronic bacterial infections of the respiratory tract and bronchiectasis, found to have a homozygous mutation in the TAP2 gene (1, 2). The transporter associated with antigen processing (TAP) is a heterodimer (TAP1 and TAP2 subunits) inserted into the membrane of the endoplasmic reticulum (ER) that transports endogenous peptides from the cytosol into the ER lumen, where they are loaded onto newly synthesized HLA class I molecules. Peptide acquisition is necessary for their stabilization, export from the ER and migration to the cell surface (3). Consequently, in the absence of a functional TAP, these processes do not efficiently occur.

In 1994, it was already established, in the context of the “missing self-hypothesis” formulated by Ljunggren and Kärre, that natural killer (NK) cells, the third type of lymphocytes besides B and T cells, preferentially killed targets not expressing HLA class I molecules (4). However, peripheral blood mononuclear cells from the TAP-deficient patients (containing the NK cell fraction), which contained a normal percentage of NK cells compared to healthy donors, were not cytotoxic to HLA class I negative K562 leukemia cells (1). Mouse models of TAP1- and β2-microglobulin (β2m) deficiency confirmed the observation, as NK cells from these animals were likewise hypo-responsive (5, 6).

## NK Cells From TAP-deficient Patients Express Inhibitory NK Cell Receptors

The project for my master thesis aimed at studying NK cells from the two TAP-deficient patients in more detail, as Alessandro Moretta previously had described several monoclonal anti-human antibodies of murine origin that identified NK cell subpopulations and whose target molecules were clonally distributed (indeed, the Moretta lab was successful in cloning NK cells) (7–9). This work later revealed that the structures recognized by the antibodies were inhibitory NK cell receptors (IR), able to negatively regulate NK cell functions.

Henri de la Salle asked Alessandro Moretta for these antibodies (GL183, EB6, Z27, and XA185), which were not yet commercially available, and received generous amounts of hybridoma cell culture supernatants in the context of a scientific collaboration. My role was first to check if the patient's NK cells, drawn from peripheral blood, did express the IR recognized by the antibodies. We did not purify the NK cells but identified them by three-color flow cytometry after staining with anti-CD3 (specific T cell marker), anti-CD19 (specific B cell marker), and anti-IR antibodies. Natural killer cells were considered as the CD3-CD19- events in the lymphocyte gate which allowed to evaluate the percentages of cells positive for the different IR. As controls, we used PBMC from the unaffected father of the patients (heterozygous for the TAP mutation) in addition to those from 10 unrelated healthy donors. The results clearly showed that the percentages of IR+ NK cells were in the same range for the patients, their father and the healthy donors (10), of course by taking into consideration the substantial inter-individual variability in IR expression. Patient's NK cells were no outliers, although the percentages of cells positive for the antibody XA185 were rather high (10).

A second readout for receptor expression in flow cytometry is the mean fluorescence intensity (MFI), which reflects the density of expression of a given molecule. Regarding this parameter, it turned out that the MFI for the Killer Immunoglobulin-like Receptors (KIR) recognized by the antibodies EB6 and Z27 was again in the normal range, whereas for GL183, the MFI was slightly higher for the NK cells from the patients and their father, suggesting rather a genetic cause than a relationship to the TAP deficiency. However, and interestingly, the MFI of XA185 was much more pronounced in the case of the patients' NK cells than in their father and the ten normal donors (10).

In the meantime, Prof. Moretta had developed additional anti-NK cell antibodies called Q66, FES172, and Z270. Whereas, the former two were directed against a KIR and an activating isoform, respectively, the latter was specific for CD94/NKG2A only and not for all CD94-bound receptors like XA185. CD94 is a chaperone protein necessary for the surface expression of different NKG2 molecules, and among them, the inhibitory receptor NKG2A (8). Again, we received aliquots of these antibodies, and they confirmed prior results in that the anti-KIR Q66 antibody and the anti-NKG2A Z270 antibody stained percentages of NK cells in the patients comparable to those of the father and the healthy volunteers, whereas the frequency of FES172+ cells was extremely low except in three healthy donors (10). Such a pattern was compatible with the previously observed fact that only one third of the analyzed donors had a subset of FES172+ NK cells. It is now clear that this receptor is an activating KIR isoform which binds to a bacteria-derived epitope on HLA-C (11). Furthermore, even if the MFI of Q66 was in the lower normal range, the one of Z270 was strongly increased on patients' NK cells (even slightly more than for XA185) (10).

Table 1 summarizes the different IR and, if applicable, their activating counterparts. The presence or absence of activating KIR isoforms depend on the KIR haplotype that an individual has inherited (12).

**Table 1:** Different anti-human NK cell antibodies with their antigens and the HLA class I ligand(s) of the antigens.

**Antibody**	**Antigen(s) current name(s)**	**Antigen(s) former name(s)**	**Ligand(s)**
EB6	KIR2DL1/S1	p58.1/p50.1	HLA-C group C2
GL183	KIR2DL2/L3/S2	p58.2/p50.2	HLA-C group C1
Z27	KIR3DL1/S1	p70/NKB1	HLA-B group Bw4
Q66	KIR3DL2	p140	HLA-A*03/HLA-A*11/HLA-F
FES172	KIR2DS4	p50.3	HLA-C alleles/HLA-F
XA185	CD94		HLA-E
Z270	CD94/NKG2A		HLA-E

*List of monoclonal antibodies generated in the Moretta lab and used for the study of NK cells in TAP-deficient patients. The molecules recognized by these antibodies are members of the killer immunoglobulin-like receptor (or KIR) family (first five cells in column 2) or of the C-type lectin superfamily (last two cells in column 2). All the receptors mentioned are IR, except p50.1, p50.2, and p50.3, which are AR*.

## First Meeting with Alessandro Moretta

The first time I had the honor and pleasure to meet Alessandro Moretta was in November 1996, at the Annual Congress of the French Society for Immunology held at the Pasteur Institute in Paris, France. Henri de la Salle was present too, and we listened to the brilliant presentation of Alessandro Moretta about the antibodies allowing to distinguish different NK cell subpopulations and clones. During the coffee break, he was literally assaulted by colleagues who wanted his antibodies. Fortunately, Henri de la Salle had fixed an appointment during the lunch break, and there I was introduced to Alessandro. I had read almost all his papers and, as lots of young researchers, thought that I was already very good in my field. Nevertheless, I was nervous when I was introduced to him, but his kindness and nonchalance together with an impressive scientific rigor, had an anxiolytic effect on me. He gave us interesting ideas to try out. Then, I had to present my poster describing my data. Alessandro followed with interest and asked a lot of challenging questions.

## Investigation of the Cytotoxicity of Patients' NK Cells

Back in Strasbourg, we continued the investigation of the cytotoxic properties of patients' non-activated NK cells by using ^51^Chromium release assays. Natural killer cells were purified after depletion of monocytes and T cells with anti-CD14 and anti-CD3 magnetic beads, respectively. They confirmed the absence of K562 killing, but in addition, antibody-dependent cellular cytotoxicity (ADCC) against rabbit antiserum-coated Raji Burkitt's lymphoma cells was also quite low compared to the NK cells from a healthy donor. Raji without antiserum was used as a negative control due to its resistance to unstimulated NK cells, and indeed neither the NK cells from the healthy donors nor those from the patients were cytolytic to this target. The EBV-transformed B lymphoblastoid cell lines (B-EBV) from the patients were killed to a substantial amount by healthy donor NK cells, fully in accordance with the “missing self” concept [(10) and unpublished data].

It had been described that the stimulation of NK cells with the cytokine interleukin (IL)-2 leads not only to their proliferation, but also to a strong increase in their cytotoxic properties toward targets already killed by their non-activated counterparts, and in addition to an extension of the spectrum of susceptible cell lines. Thus, Raji and Daudi, which both resist to *ex vivo* NK cells, are killed by activated ones (10).

The most efficient method for NK cell expansion and activation at that time was the one described by Perussia et al.: total PBMC are co-cultured with irradiated Burkitt's lymphoma Daudi feeder cells (at a 5:1 ratio) and IL-2 for 6 days, identically re-stimulated and then grown for a few additional days (13). We usually harvested the cells at day 12, purified the NK cells with anti-T cell immunomagnetic beads (as some T cells likewise expand under these conditions) and then used them in cytotoxicity assays. In preliminary experiments, we noticed that the method worked even better with B lymphoblastoid cell lines like ST-EMO derived from one of the patients. Later, we found that the best stimulator cell line was TND-3 (unpublished data), derived from a Japanese patient with a TAP1 deficiency (14). We have to date no clear explanation for the phenomenon that TAP-deficient B-EBV cell lines induce higher expansion rates than cell lines derived from healthy volunteers.

When we cultured normal and TAP-deficient PBMC under these conditions, we observed that the latter expanded much less well (10). Normal activated NK cells very significantly killed K562, Daudi and Raji and performed a strong ADCC against Raji. Interestingly, the TAP-deficient activated NK cells were likewise efficient in terms of natural cytotoxicity and ADCC against the same target cell lines, which was a bit surprising for us. We then tested the killing of autologous B-EBV cell lines from five healthy donors and from the patients. These experiments revealed that the percentage of lysis of the normal B-EBV cells never exceeded 15 %, whereas it reached 35% in the case of patients' cell lines (10). This observation suggests some degree of auto-aggressivity exerted by the activated NK cells of these individuals.

## NK Cell-Mediated Killing of TAP-deficient Fibroblasts

Having demonstrated this autologous killing performed by TAP-deficient NK cells (in the meantime I had become a PhD student), we went on to check if it was also the case against primary cells, namely skin fibroblasts. We cultured them with HLA class I-inducing cytokines (IFN-α, IFN-γ, IFN-γ + TNF-α) and used them as targets for purified activated autologous NK cells from three healthy donors and one of the patients (15). As expected, the patient's fibroblasts only marginally increased their surface HLA class I expression (W6/32 antibody), in contrast to the normal fibroblasts. As a consequence, the former were strongly lysed by their autologous NK cells, whereas healthy fibroblasts were protected either completely or at least to a significant extent. We then thought to demonstrate that the masking of HLA class I by the A6-136 antibody (IgM isotype) from the Moretta lab restored (normal cells) or increased (patient's cells) the killing of the fibroblasts This was clearly the case for the healthy fibroblasts but not for the TAP-deficient ones, for whom no effect could be observed (15).

In the next step, T lymphoblasts from phytohemagglutinin (PHA)-stimulated cultures were used as targets. When originating from healthy donors exposed to autologous activated NK cells, almost no lysis was observed, whereas such NK cells efficiently killed TAP-deficient PHA-blasts. The latter were surprisingly only killed by healthy volunteers' NK cells but not by autologous patients' NK cells, in contrast to our previous observations with B-EBV lymphoblastoid cell lines and skin fibroblasts. When the A6-136 antibody was added to the cytotoxicity assays, autologous T-PHA blasts from the healthy donors were significantly lysed by their autologous activated NK cells. Inversely, the patients' activated NK cells did not attack autologous T-PHA blasts.

During all this work, Henri and me regularly discussed the data by phone (Skype didn't yet exist) with Alessandro who was very interested in the topic and gave useful advice. To facilitate the exchanges, I traveled twice to his lab where I received a very warm welcome from him and his collaborators. We shipped PBMC and fibroblasts to Genova to be able to do further experiments there. I still precisely remember the scientific discussions we had at these occasions, because they represented high quality lessons in immunology during which I learned a lot. On the last evening of my stays, Alessandro took us to nice restaurants where I became aware even more what a fascinating personality Alessandro had and where I was delighted to enjoy local pasta specialties in a lot of different variations.

## Expression of Activating Receptors

It had become clear that NK cells need not only IR, but also activating receptors (AR) to efficiently kill targets. Here again, Alessandro was the first to identify a family of them, namely the natural cytotoxicity receptors (NCR) NKp30, NKp44, and NKp46 (16). We received the corresponding antibodies before their appearance on the market and could show that these molecules were expressed totally normally by the NK cells of the patients. Regarding NKG2D, Alessandro was scooped by another group (from whom we could not obtain an antibody aliquot), although his lab later developed antibodies against this AR, too. It is likewise expressed by TAP-deficient NK cells.

The work in Alessandro's lab further allowed to demonstrate that TAP-deficient activated NK cells are able to produce IFN-γ but difficult to clone (17), and we completed together the phenotypic studies of our patients in 2007, focusing this time on the expanded CD56bright population and the overexpression of the broad-spectrum IR, NKG2A, and ILT2 (18).

## Further Cases of TAP Deficiency

Other groups identified TAP-deficient patients as well and overall confirmed our data (19, 20). We then described a patient with cytotoxic NK cells at the baseline (21). This individual was heavily infected in the airways at the time of blood drawing, and thus her NK cells might have been activated *in vivo*. Over the years, several additional patient observations appeared in the literature, focusing more on the clinical aspects (22–29). Based on the number of published cases, TAP deficiency is extremely rare, but there are probably much more patients, as the frequent diagnosis of idiopathic bronchiectasis only seldomly leads to a check for normal HLA class I expression (30), and as the diagnosis is probably often not made in regions with a difficult access to medical structures and a high consanguinity rate.

The last time I met Alessandro was in 2012, when I organized a scientific meeting in Luxembourg, entitled “HLA class I molecules in Health and Disease,” bringing together an impressive international speaker panel composed of first-class immunologists. There was a session exclusively dedicated to TAP deficiency, whereas Alessandro gave a talk about his current work. For the gala dinner, I arranged to sit at his table, and once more, we had very interesting and stimulating discussions.

## Summary and Outlook

In the light of the vertiginous evolution Immunology has taken in recent years, can we give a new interpretation to our findings about TAP deficiency? It is now obvious that NK cells from TAP-deficient patients are not educated (not licensed), but Henri de la Salle and myself were not creative enough to go deeper into the hyporesponsiveness mechanisms of these cells, and we also did not resolve the molecular basis of the cytotoxic activity after cytokine stimulation.

Most of the patients described to date have serious and chronic bacterial infections of the respiratory tract (leading to bronchiectasis), whereas viral infections of course occur but are not exceptionally severe (3). In addition, most of them also suffer from deep skin ulcers on the legs that are healing very slowly and are difficult to treat (3, 27, 29). They might even develop granulomatous lesions of the nose, leading to the complete destruction of the nasal cartilage. It is known that NK cells are involved in antibacterial defense but that their effects can be beneficial as well as deleterious, depending on the pathogen and the precise context (31–33). Natural killer cells in tissues are different, at least in part, from those circulating in peripheral blood (34). Therefore, we cannot say for the moment if lung NK cells from the patients are dysfunctional in terms of antibacterial defense and/or if they destroy bronchial tissues due to their hypothetical (over-)activation in the infectious situation. Likewise, skin NK cells might be at the origin of the skin ulcers, where they have indeed been found (19). The nasal lesions resemble lethal midline granuloma which is now called NK/T cell lymphoma, nasal type, and this suggests that, once activated, NK cells destroy autologous tissues which in the case of TAP deficiency do not express sufficient amounts of HLA class I molecules to inhibit these cytolytic effectors. Recently, we found that TAP-deficient patients have a higher percentage of the newly described CD56dimCD16dim NK cell subset than healthy controls (35), and this might be the result of a slightly delayed maturation of patients' NK cells. The Malmberg group, in collaboration with our lab, also showed that a polyclonal expansion of adaptive NKG2C+ NK cells occurs in the patients, which might partially explain their resistance to severe viral infections (36). Furthermore, we analyzed in detail the repertoire of the HLA class I-binding receptors KIR, NKG2A and CD8 and found a correlation between the presence/absence of HLA class I molecules and the coexpression of their receptors (37).

In summary, Alessandro Moretta has made substantial contributions to the in-depth description of TAP-deficient NK cells. Without his antibodies, I might not have been able to publish in high quality journals during my PhD thesis and to find a postdoc position in the lab of Prof. Werner Held at the Ludwig Institute in Lausanne, Switzerland. Interestingly, Alessandro had started his career at this same Institute, in the lab of his brother Lorenzo.

His work has not only theoretical but also practical clinical implications, as illustrated by the well-known phenomenon of KIR-ligand mismatch in semi-allogeneic bone marrow grafts (38). Very recently, a NK cell engager targeting NKp46, CD16 and a tumor antigen was described by the Vivier group (39), and this tri-specific engager holds quite some promise for cancer immunotherapy.

Alessandro Moretta was an outstanding scientist and a very nice person. I am sad that we lost him, but I am proud and honored to have known him.

## Data Availability Statement

All datasets generated for this study are included in the manuscript/supplementary files.

## Author Contributions

JZ conceived and wrote the article.

### Conflict of Interest

The author declares that the research was conducted in the absence of any commercial or financial relationships that could be construed as a potential conflict of interest.
